# Identification of potentially functional circRNAs and prediction of the circRNA-miRNA-hub gene network in mice with primary blast lung injury

**DOI:** 10.1186/s12890-023-02717-9

**Published:** 2023-10-28

**Authors:** Qianying Lu, Junfeng Li, Yanmei Zhao, Jianfeng Zhang, Mingyu Shi, Sifan Yu, Yangfan Liang, Haojun Fan, Xiangyan Meng

**Affiliations:** 1 Institute of Disaster and Emergency Medicine, Tianjin University, No. 92, Weijin Road, Nankai District, Tianjin, 300072 China; 2 Tianjin Key Laboratory of Disaster Medicine Technology, No. 92, Weijin Road, Nankai District, Tianjin, 300072 China; 3 Wenzhou Safety (Emergency) Institute, Tianjin University, Wenzhou, 325000 China

**Keywords:** Blast, Lung, circRNAs, circRNA-miRNA-hub gene network, Therapy

## Abstract

**Objectives:**

Primary blast lung injury (PBLI) is the main cause of death in blast injury patients, and is often ignored due to the absence of a specific diagnosis. Circular RNAs (circRNAs) are becoming recognized as new regulators of various diseases, but the role of circRNAs in PBLI remain largely unknown. This study aimed to investigate PBLI-related circRNAs and their probable roles as new regulators in PBLI in order to provide new ideas for PBLI diagnosis and treatment.

**Methods:**

The differentially expressed (DE) circRNA and mRNA profiles were screened by transcriptome high-throughput sequencing and validated by quantitative real-time PCR (qRT-PCR). The GO and KEGG pathway enrichment was used to investigate the potential function of DE mRNAs. The interactions between proteins were analyzed using the STRING database and hub genes were identified using the MCODE plugin. Then, Cytoscape software was used to illustrate the circRNA-miRNA-hub gene network.

**Results:**

A total of 117 circRNAs and 681 mRNAs were aberrantly expressed in PBLI, including 64 up-regulated and 53 down-regulated circRNAs, and 315 up-regulated and 366 down-regulated mRNAs. GO and KEGG analysis revealed that the DE mRNAs might be involved in the TNF signaling pathway and Fanconi anemia pathway. Hub genes, including Cenpf, Ndc80, Cdk1, Aurkb, Ttk, Aspm, Ccnb1, Kif11, Bub1 and Top2a, were obtained using the MCODE plugin. The network consist of 6 circRNAs (chr18:21008725–21020999 + , chr4:44893533–44895989 + , chr4:56899026–56910247-, chr5:123709382–123719528-, chr9:108528589–108544977 + and chr15:93452117–93465245 +), 7 miRNAs (mmu-miR-3058-5p, mmu-miR-3063-5p, mmu-miR-668-5p, mmu-miR-7038-3p, mmu-miR-761, mmu-miR-7673-5p and mmu-miR-9-5p) and 6 mRNAs (Aspm, Aurkb, Bub1, Cdk1, Cenpf and Top2a).

**Conclusions:**

This study examined a circRNA-miRNA-hub gene regulatory network associated with PBLI and explored the potential functions of circRNAs in the network for the first time. Six circRNAs in the circRNA-miRNA-hub gene regulatory network, including chr18:21008725–21020999 + , chr4:44893533–44895989 + , chr4:56899026–56910247-, chr5:123709382–123719528-, chr9:108528589–108544977 + and chr15:93452117–93465245 + may play an essential role in PBLI.

## Introduction

Blast injuries are the most common fatal injuries in military actions, terrorist attacks, and peacetime accidents, such as industrial accidents and gas explosions [1]. In recent years, explosion accidents have occurred frequently, and blast injury has attracted increasing attention [2, 3]. At the moment of the explosion, the power is rapidly released in the form of heat, gas products and shock waves. Among these forms of power, shock waves are an important cause of injury, and can cause damage to multiple systems and organs, namely, primary blast injury [4]. The lung is the most vulnerable target organ in blast injury due to its air-containing. Clinical reports show that exposure to blast shock waves can easily cause primary blast lung injury (PBLI), leading to severe clinical manifestations, such as severe pulmonary contusion, hemorrhage, edema, and further acute lung injury (ALI), even more serious acute respiratory distress syndrome (ARDS), which eventually leads to death [5–8]. PBLI is the main cause of death for blast injury patients [9]. However, because of the lack of a precise diagnosis, there is a contradiction between the low diagnosis rate and high morbidity and mortality in PBLI patients.

CircRNAs are a class of non-coding RNAs (ncRNAs) with a covalently closed loop structure. With the development of high-throughput sequencing technology, an increasing number of circRNAs have been identified. It has been reported that circRNAs are involved in several diseases, such as cardiovascular disease, digestive system disease and cancers [10–12]. CircRNAs exert important biological functions by acting as microRNA (miRNA) sponges, interacting with proteins or being translated themselves [13]. Accumulating evidence indicates that circRNAs involved in diseases mainly act as competing endogenous RNAs (ceRNAs), or miRNA sponges, to adsorb miRNAs as miRNA response elements (MREs), which indirectly regulate the expression of downstream miRNA target genes [14]. The ceRNA network may serve a role in the pathophysiology and treatment of diseases. However, data on circRNAs and circRNA-associated ceRNA networks in PBLI are largely absent. Therefore, there is an urgent need to explore circRNAs and circRNA-related ceRNA networks in PBLI.

Advances in bioinformatics offer new opportunities to advance the understanding of circRNAs and circRNA-related ceRNA networks in diseases. For example, GO and KEGG analysis are two commonly used methods in bioinformatics that link genes and functions together [15, 16]. Jin et al. [17] performed GO and KEGG analysis to analyze the potential biological function of DE genes in nonalcoholic steatohepatitis, which may provide novel mechanism for nonalcoholic steatohepatitis. And many tools have been designed for miRNA target prediction. For example, miRanda and TargetScan databases are widely used databases that predict biological targets of miRNAs based on the principle of sequence complementarity. Deng et al. [18] used the miRanda and TargetScan databases to predict miRNA binding seed sequence sites, and constructed ceRNA networks, to provide new insights into the pathogenesis of AD. And Su et al. [19] used the miRanda and TargetScan databases to explore circRNA-related ceRNA network in pediatric pulmonary hypertension linked congenital heart disease. Therefore, bioinformatics can help us better understand the role of circRNAs and ceRNA networks in diseases. In addition, some computational models have also been used to predict the circRNA-disease association, and the establishment of these models relies on existing databases. However, the data of PBLI or circRNAs related to PBLI are almost absent.

**Fig. 1:**
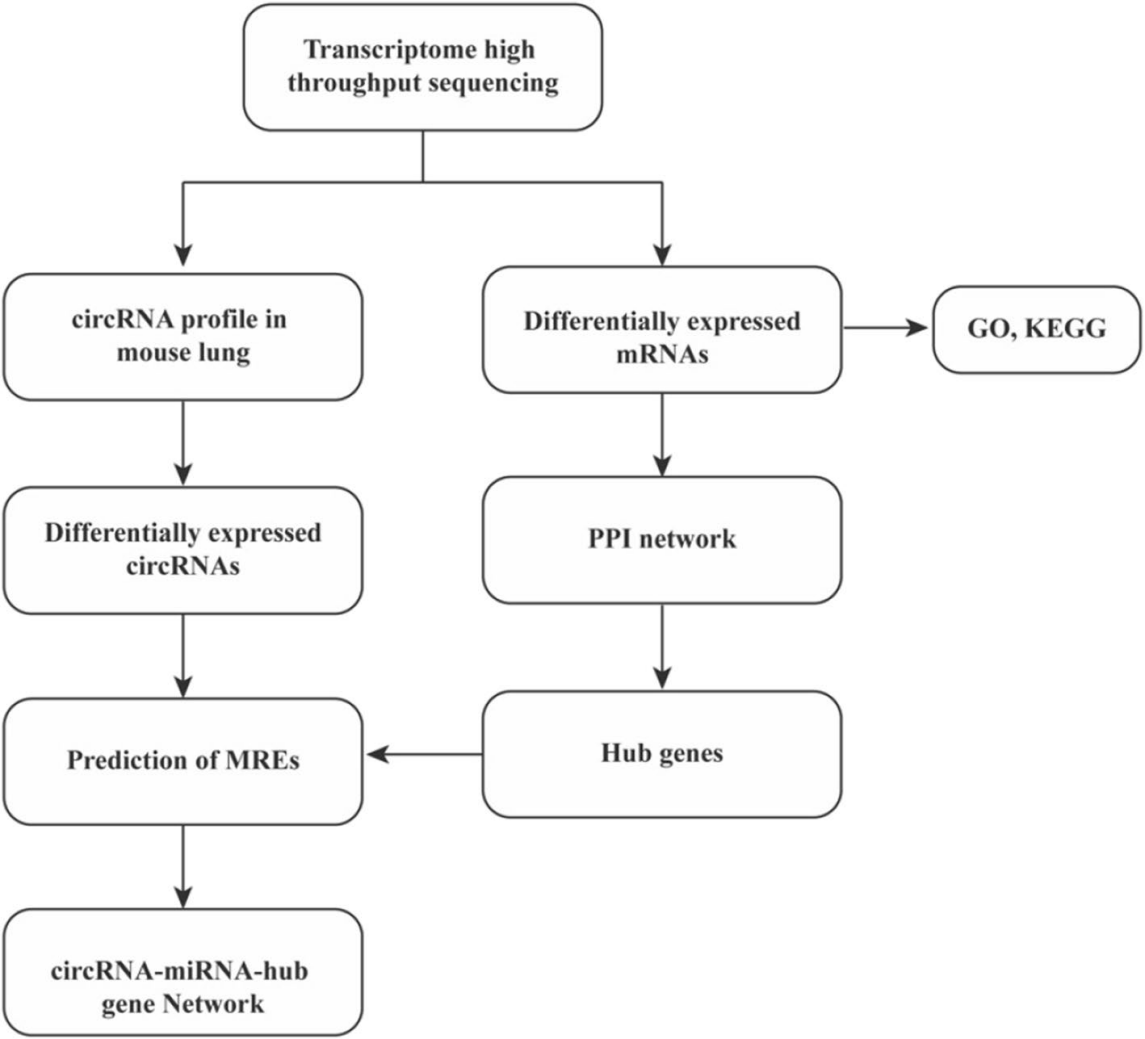
Flow chart of the present study. CircRNA circular RNA, GO Gene Ontology, KEGG Kyoto Encyclopedia of Genes and Genomes, PPI protein–protein interaction, MREs microRNA response elements

In this study, we used high-throughput sequencing techniques and bioinformatics methods to investigate the regulatory mechanism of circRNAs in PBLI, especially the circRNA-miRNA-hub gene network for the first time (Fig. 1). Our findings would provide new evidence for understanding the molecular mechanisms of circRNAs in the PBLI and for developing improved diagnosis and treatment strategies for PBLI.

## Methods

### Experimental animals

Male C57BL/6 mice, aged 8–10 weeks and weighing 20–22 g, were purchased from Beijing Vital River Laboratory Animal Technology Co., Ltd. (Beijiing, China). All mice were housed in a temperature-controlled and specific-pathogen-free environment with a 12 h light/dark cycle. All mice were free access to food and water. And all experimental procedures and protocols were conducted in accordance with the guidelines of the local animal care and use committee. Animal welfare and experimental design were approved by the Institutional Animal Care and Use Committee of Yi Shengyuan Gene Technology (Tianjin) (YSY-DWLL-2021013). The study is reported in accordance with ARRIVE guidelines.

### Establishment of primary blast lung injury model in mice

The self-made mini shock tube stimulation device was used to establish the PBLI model as previously described [20]. In brief, twelve mice were randomly divided into two groups: the control group and the PBLI group. For statistical analysis, six mice were included in each group. After anesthesia, the mice in PBLI group were placed in a prone position on a black rubber plate that attached to the device, with holes in the rubber plate located directly below the mice's chest. A shock wave with a peak pressure of 0.5 bar was generated using the device to cause lung injury in mice. The animals in the control group were anesthetized in the same manner but were not exposed to blast overpressure.

### Sample preparation

Six hours post-blast, euthanasia by cervical dislocation was performed and the lung of all mice was harvested. The right upper lobe of lungs was taken and placed in enzyme-free EP tubes, part of lung tissue of each mouse was cut and stored in liquid nitrogen for subsequent sequencing. The other part was added with 1 mL TRIzol reagent, homogenated and placed in the refrigerator at -80℃ for the RNA extraction. Every two tissues used for sequencing were mixed into one sample, that is, 3 samples each in the control group and the PBLI group for sequencing.

### Total RNA isolation, library construction and sequencing

Total RNA from the lung tissue of the two groups of mice was isolated using TRIzol reagent (Invitrogen). The concentrations and purity of the RNA were measured using a NanoDrop ND-1000 instrument (Thermo, Waltham, MA, USA). OD260/OD280 values were used as the purity index of RNA, and OD260/OD280 values ranging from 1.8 to 2.1 were considered to be qualified. And RNA integrity and gDNA contamination were measured using denatured agarose gel electrophoresis.

The transcriptome high-throughput sequencing service was provided by CloudSeq Biotech (Shanghai, China). Briefly, rRNAs in total RNA were removed using the NEBNext® rRNA Depletion Kit (New England Biolabs, Inc., Massachusetts, USA) following the manufacturer's instructions. The TruSeq Stranded Total RNA Library Prep Kit (Illumina, USA) was used to construct RNA sequencing libraries with RNA-depleted RNAs. The BioAnalyzer 2100 instrument (Agilent Technologies, USA) was used to perform the library quality control and quantification. The 150 bp double-ended reads sequencing was performed on the Illumina NovaSeq 6000 instrument.

### Data quality control

After image and base recognition, double-ended raw reads were harvested from the Illumina NovaSeq 6000 sequencer. Q30 was used for quality control, cutadapt software (v1.9.3) was used to remove connectors, low-quality reads were removed, and high-quality clean reads were obtained.

### Differential expression of circRNAs in the lungs of PBLI mice

The high-quality reads were aligned to the reference genome/transcriptome with STAR software (v2.5.1b) and circRNAs were detected and identified with DCC software (v0.4.4). According to the alignment position of two ends of circRNAs, the circRNAs were divided into exonic circRNAs, intronic circRNAs, or inter-geneic circRNAs. The circRNAs were then identified using the circBase database and circ2Trait circRNA-disease database links based on the circRNA genome location. The newly identified circRNAs were labeled as novel.

The default TMM method of edgeR software (v3.16.5) was used to standardize the original junction reads, and the number of standardized reads was used to calculate the differential expression of circRNAs between the two groups. A fold change ≥ 2.0 and a *p* value ≤ 0.05 were taken as the threshold for DE circRNAs.

### Differential expression of mRNAs in the lungs of PBLI mice

The high-quality reads were aligned to the mouse reference genome (UCSC MM10) with hisat2 software (v2.0.4). Then, FPKM values were obtained to create an expression profile of mRNA using cuffdiff software (v2.2.1, part of cufflinks) under the guidance of the Ensembl gtf gene annotation file. The cuffdiff software was used to identify DE mRNAs between the two groups. A fold change ≥ 2.0, *p* value ≤ 0.05, and FPKM value ≥ 0.5 in at least one sample were considered as differentially expressed.

### Validation and quantification of circRNAs

The DE circRNAs were validated by quantitative real-time PCR (qRT-PCR). Two upregulated and two downregulated circRNAs, and three circRNAs in the ceRNA network were randomly selected. The housekeeping gene β-actin was used as a reference for normalization, and the primers for circRNAs were specific divergent primers (Table 1). Total RNA was reverse transcribed into complementary DNA using the PrimeScript RT Reagent Kit (Perfect Real Time; TaKaRa, Osaka, Japan), and qRT-PCR was performed on a Roche LightCycler 96 Real-Time PCR System using the Hieff® qPCR SYBR Green Master Mix (Yeasen, Shanghai, China) according to the manufacturer’s instructions. Three independent assays were performed on all samples, in which samples were assessed in triplicate. The 2^−ΔΔCt^ method was used to calculate the relative expression of the circRNAs.

**Table 1:** Primers designed for qRT-PCR validation of selected circRNAs

circRNA name	Forward primer	Reverse primer
chr13:117249272–117249786 +	TGGGCAACACCTTAACCAGT	ACATTGGACTTTTCGTGGATGT
chr2:74568941–74573626-	TGCAAAAATTATGGGTTGGA	TTGCAGAAACTTGGCACAAT
chr3:122794260–122804609 +	CATCTTCTGTGGCTTGGGGAT	TCTTGCTCCCGATAAAGCCTG
chr2:128675671–128676247-	TCCATTCACAGCCAGAGTCG	AAAAAGACCTTAGAGCCGCCA
chr18:21008725–21020999 +	GGATACAAGTTCTTCTGGGCA	CACACTTCCACGACATAGGG
chr4:44893533–44895989 +	CGTTACTGCTTAGACTGCTCT	CTTGGCCTCGATGTCTTTGT
chr4:56899026–56910247-	GCGATCAGTTGGCTTCCTGAG	ATCACAGCCGTATTGTTCACCT
β-actin	AGTGTGACGTTGACATCCGT	GCAGCTCAGTAACAGTCCGC

### Functional analysis

To better understand the pathogenesis of PBLI, we used Gene Ontology (GO) (http://www.geneontology.org/) analysis and Kyoto Encyclopedia of Genes and Genomes (KEGG) (http://www.genome.jp/kegg) pathway analysis to predict the potential function of linear transcripts of DE mRNAs [21–23]. GO analysis consists of three components: Molecular Function (MF), Biological Process (BP), and Cell Component (CC). A *p* value ≤ 0.05 in GO terms is considered statistically significant. The KEGG pathway analysis is the process of mapping molecular data sets from genomics, transcriptomics, proteomics, and metabolomics onto the KEGG pathway map for the biological function interpretation of these molecules. A *p* value ≤ 0.05 was considered to indicate significant enrichment.

### Construction of the PPI regulatory network and screening of hub genes

The STRING database (http://string-db.org) was used to predict the interaction of proteins, the minimum required interaction score was set to high confidence (0.7), and the PPI network was established by CytoScape 3.9.1 software, an open source software platform for visualizing complex networks and integrating these with any type of attribute data. The MCODE plug-in of Cytoscape software, a clustering algorithm that reclassifies existing proteins or genes, was run to predict the most significantly meaningful protein modules. The advanced options were set as degree cutoff = 2, haircut, node score cutoff = 0.2, k-core = 2, and max depth = 100 [24]. In addition, hub genes in the PPI network were filtered by the Maximal Clique Centrality (MCC) arithmetic of the Cytoscape plug-in cytoHubba, which ranks nodes according to their attributes in the network, mining key genes (hub genes) and sub networks [25, 26], the top ten nodes ranked by the MCC algorithm were considered as hub genes.

### Prediction of circRNA-miRNA-hub gene associations

CircRNA-miRNA and miRNA-hub gene interactions were predicted by miRanda (http://www.miranda.org/) and TargetScan (http://www.targetscan.org/vert_71/), widely used databases that predict biological targets of miRNAs. For each circRNA/mRNA, the top 5 miRNAs that potentially bind are listed. Upregulated circRNA-miRNA-upregulated mRNA, and downregulated circRNA-miRNA-downregulated mRNA were selected, and the network was constructed by Cytoscape 3.9.1 software.

## Results

### Identification of circRNAs in mouse lung tissue

**Fig. 2:**
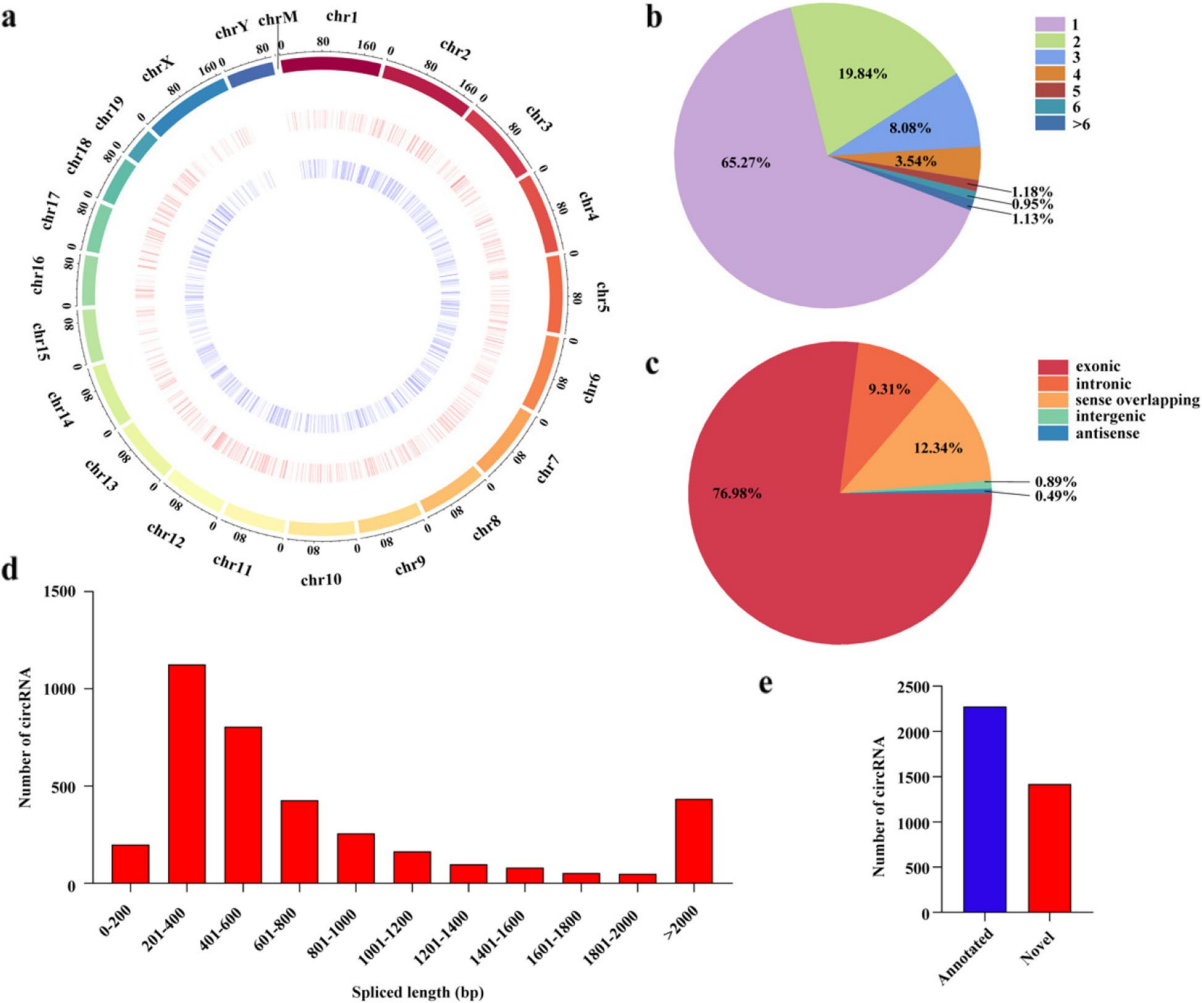
Expression patterns of circRNAs in mouse lungs. **a** Chromosome distribution of the 3696 circRNAs identified in the two groups. **b** Number of circRNAs produced from one host gene. **c** Percentages of circRNAs arising from different genomic loci. **d** Length distributions of the identified circRNAs. **e** Sources of the annotated circRNAs

In the present study, a total of 3696 circRNAs were detected from the control group and the PBLI group. These circRNAs were located in the whole genomic region (Fig. 2a), including chrX (2.30%), chrY (0.14%), and chrM (0.11%). A total of 34.73% of host genes could produce two or more circRNAs, and some genes could generate more than ten circRNAs (0.14%) (Fig. 2b). For example, Ahank, a gene located on chr19, produced 29 circular intronic RNAs, which was the most in this study. The circRNAs found so far mainly arose from exons, accounting for 76.98% of the circRNAs in this study (Fig. 2c). Sequence length analysis revealed that the circRNA transcripts were mainly 200–1200 bp in length (75.24%) but that 11.74% were over 2000 bp in length (Fig. 2d). Among the 3696 circRNAs that were identified in this study, 2276 circRNAs were already recorded in the circBase database or PubMed, and 1420 were considered as novel (Fig. 2e).

### Identification and validation of DE circRNAs in PBLI in lung tissue

**Fig. 3:**
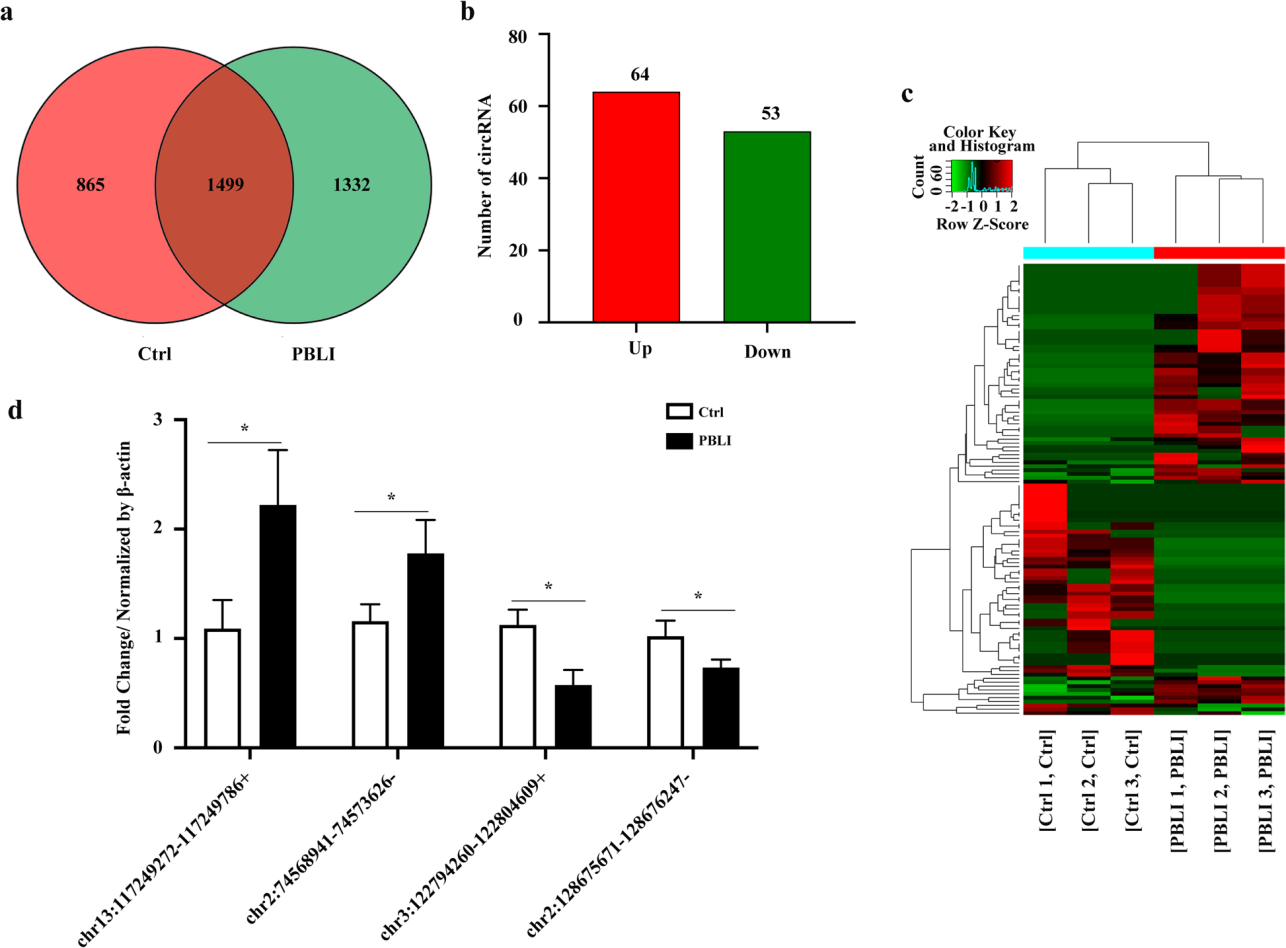
Identification and validation of DE circRNAs. **a** Numbers of circRNAs detected in the two groups. **b-c** Histogram (**b**) and Heatmap (**c**) showing the significantly up-regulated and down-regulated circRNAs in the PBLI group. **d** Validation of differentially expressed circRNAs by qRT-PCR. The results are presented as the mean ± SD. *P < 0.05

To investigate the possible circRNAs and mRNAs involved in PBLI, the DE circRNAs and mRNAs between the two groups were analyzed. Among the 3696 circRNAs identified in this study, 865 were detected only in the control group, 1332 were detected only in the PBLI group, and 1499 were detected in both groups (Fig. 3a). A total of 117 circRNAs were aberrantly expressed in the PBLI group, including 64 up-regulated and 53 down-regulated circRNAs (Fig. 3b, c). The top 20 up-regulated and down-regulated circRNAs are shown in Table 2. Two up-regulated (chr13:117249272–117249786 + and chr2:74568941–74573626-) and two down-regulated circRNAs (chr3:122794260–122804609 + and chr2:128675671–128676247-) were randomly selected for validation of DE circRNAs, which showed that the expression levels of DE circRNAs were consistent with the sequencing results (Fig. 3d).

**Table 2:** Top 20 significantly up- and down-regulated circRNAs

CircRNAID	logFC	*P* Value	FDR	Regulation	Chrom	Strand	CircRNA type	GeneSymbol
chr14:74807474–74835775-	4.780316216	0.007914504	0.549906578	up	chr14	-	exonic	Lrch1
chr7:90160448–90165616 +	4.7414159	0.0088942	0.5499066	up	chr7	+	exonic	Picalm
chr9:18295300–18302183 +	4.7412335	0.0081513	0.5499066	up	Chr9	-	exonic	Chordc1
chr2:120806759–120825315-	4.6265529	0.0102995	0.5499066	up	Chr2	-	exonic	Ttbk2
chr10:105413217–105413787-	4.3831007	0.0174558	0.5499066	up	Chr10	-	exonic	Tmtc2
chr10:67157907–67189849 +	4.318324	0.0197947	0.5499066	up	Chr10	+	exonic	Jmjd1c
chr4:137604523–137605908 +	4.2040936	0.0266802	0.5499066	up	Chr4	+	exonic	Usp48
chr1:53904249–53914632-	4.2017178	0.0268239	0.5499066	up	Chr1	-	exonic	Hecw2
chr5:138616566–138617054-	4.1900263	0.027293	0.5499066	up	Chr5	-	intronic	Zfp68
chr10:42196466–42197901-	4.1734726	0.0280674	0.5499066	up	Chr10	-	exonic	Foxo3
chr19:56570416–56576284 +	4.1449804	0.0293986	0.5499066	up	Chr19	+	exonic	Nhlrc2
chr10:59166504–59178857-	4.1437402	0.0294798	0.5499066	up	Chr10	-	exonic	Sept10
chr18:80869742–80917878-	4.0311917	0.0396353	0.5499066	up	Chr18	-	exonic	Atp9b
chr5:128268492–128269377-	4.0311917	0.0396353	0.5499066	up	Chr5	-	exonic	Tmem132d
chr17:66436080–66438391-	3.9977349	0.041233	0.5499066	up	Chr17	-	exonic	Mtcl1
chr5:28358274–28394670 +	3.9964495	0.041395	0.5499066	up	Chr5	+	exonic	Rbm33
chr2:119076936–119081625 +	3.9964495	0.041395	0.5499066	up	Chr2	+	exonic	Casc5
chr11:61532518–61535383-	3.9964495	0.041395	0.5499066	up	Chr11	-	exonic	Epn2
chr14:46762528–46768897 +	3.9809425	0.0413481	0.5499066	up	Chr14	+	exonic	Cdkn3
chr16:32027712–32037308-	3.9783187	0.0416747	0.5499066	up	Chr16	-	exonic	Pak2
chr4:11218612–11219926-	-5.2966588	0.0006025	0.5499066	down	chr4	-	exonic	Ints8
chr17:86098512–86120746-	-4.4340689	0.0170265	0.5499066	down	chr17	-	exonic	Srbd1
chr4:56899026–56910247-	-4.4246127	0.0173766	0.5499066	down	chr4	-	exonic	Tmem245
chr17:71273374–71275287-	-4.2400633	0.021767	0.5499066	down	chr17	-	exonic	Emilin2
chr5:123709382–123719528-	-4.2197784	0.0225342	0.5499066	down	chr5	-	sense overlapping	Zcchc8
chr4:44893533–44895989 +	-4.2173542	0.0226263	0.5499066	down	chr4	+	exonic	Zcchc7
chr18:21008725–21020999 +	-4.2173542	0.0226263	0.5499066	down	chr18	+	exonic	Rnf138
chr2:128675671–128676247-	-4.2161414	0.0226724	0.5499066	down	chr2	-	exonic	Anapc1
chr9:108528589–108544977 +	-4.210192	0.022903	0.5499066	down	chr9	+	exonic	Qrich1
chr6:136634431–136641214-	-4.2089763	0.0229496	0.5499066	down	chr6	-	exonic	Plbd1
chr2:18906229–18908152-	-4.2005505	0.0233045	0.5499066	down	chr2	-	exonic	Pip4k2a
chr11:98263470–98267858 +	-4.190718	0.0238582	0.5499066	down	chr11	+	exonic	Cdk12
chr3:57769040–57796295 +	-4.1820027	0.0243545	0.5499066	down	chr3	+	exonic	Rnf13
chr3:152420015–152458562 +	-3.9956976	0.0372379	0.5499066	down	chr3	+	sense overlapping	Zzz3
chr1:60268012–60292964 +	-3.9956976	0.0372379	0.5499066	down	chr1	+	sense overlapping	Nbeal1
chr4:129838978–129842762 +	-3.9563162	0.0390072	0.5499066	down	chr4	+	exonic	Ptp4a2
chr19:36961639–36970038 +	-3.9413751	0.0397058	0.5499066	down	chr19	+	exonic	Btaf1
chr14:20515523–20531860-	-3.9413751	0.0397058	0.5499066	down	chr14	-	exonic	Ppp3cb
chr8:82762967–82763263-	-3.9297549	0.0402686	0.5499066	down	chr8	-	intergenic	
chr3:122760878–122795321 +	-3.9297549	0.0402686	0.5499066	down	chr3	+	exonic	Pde5a

### Identification of DE mRNAs in PBLI

**Fig. 4:**
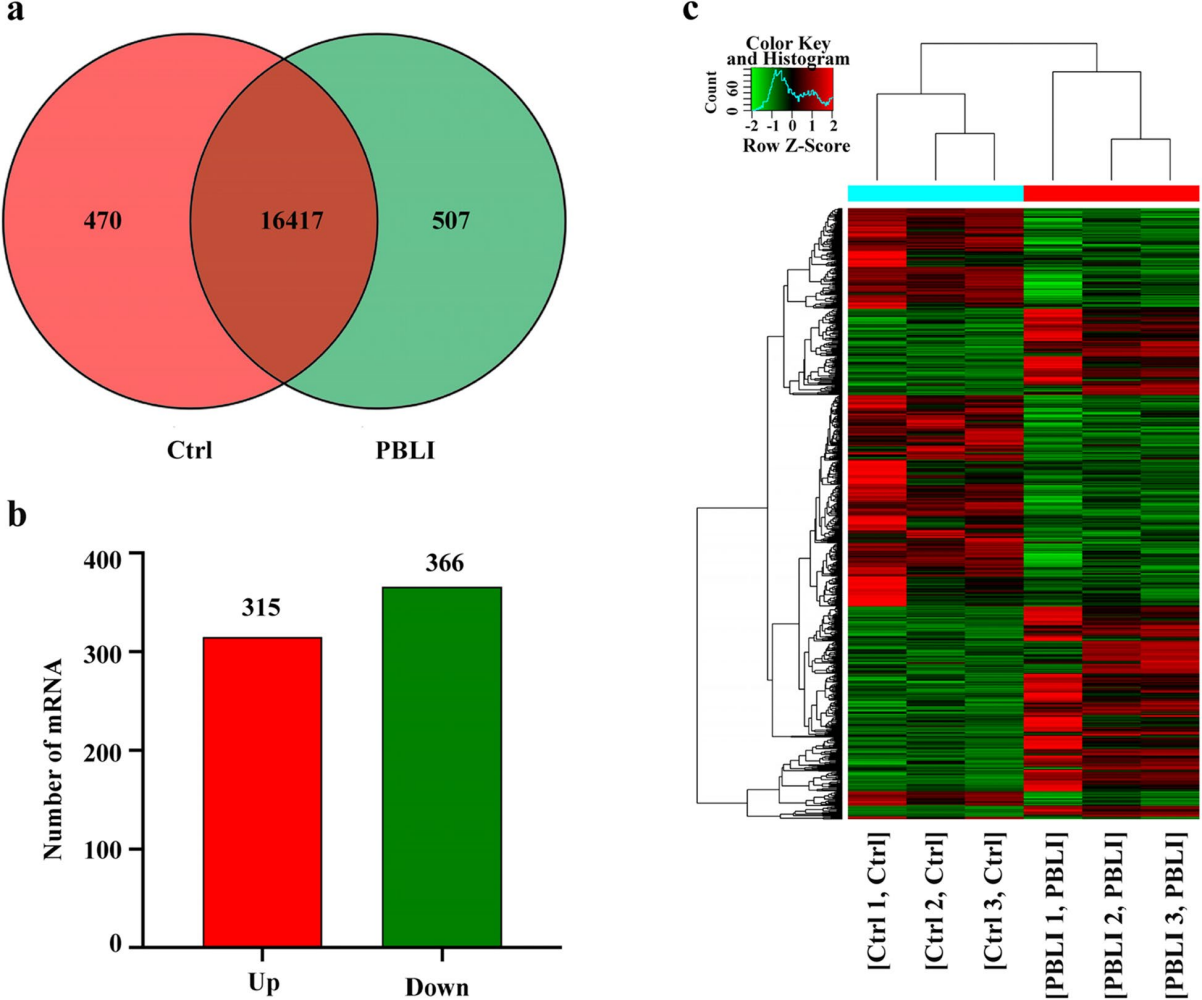
Identification of DE mRNAs. **a** Numbers of mRNAs detected in the two groups. **b-c** Histogram (**b**) and Heatmap (**c**) showing the significantly up-regulated and down-regulated mRNAs in the PBLI group

A total of 17,453 mRNAs were identified in the two groups, of which 470 were expressed only in the control group, 507 were expressed only in the PBLI group, and 16,417 were expressed in both groups (Fig. 4a). As shown in Fig. 4b and c, 681 mRNAs were aberrantly expressed in the PBLI group, among them, 315 were up-regulated and 366 were down-regulated. The top 20 up-regulated and down-regulated mRNAs were shown in Table 3.

**Table 3:** Top 20 significantly up- and down-regulated mRNAs

Gene	Locus	PBLI_ FPKM	Control_FPKM	Fold Change	*p*_value	FDR	Regulation
Fosl1	chr19:5447702–5455945	5.27731	0	inf	0.00005	0.00154923	up
Ccl20	chr1:83116765–83119167	1.57929	0	inf	0.00005	0.00154923	up
Prok2	chr6:99711298–99732553	1.09838	0	inf	0.00005	0.00154923	up
Prss22	chr17:23993534–23998100	1.12329	0	inf	0.00005	0.00154923	up
Gjb4	chr4:127325234–127368735	1.02015	0	inf	0.03615	0.255144	up
Gsta1	chr9:78230655–78242684	3.40052	0	inf	0.00005	0.00154923	up
Defb25	chr2:152622355–152623053	1.74469	0	inf	0.02285	0.188756	up
Sprr1a	chr3:92483951–92485895	13.3429	0.273943	48.70709382	0.00335	0.047983	up
Slc7a11	chr3:50364935–50443613	33.3669	0.955485	34.92141228	0.00005	0.00154923	up
Mt2	chr8:94170751–94173567	276.153	13.451	20.5302601	0.00005	0.00154923	up
Msln	chr17:25748613–25754327	2.72549	0.178549	15.26472824	0.00005	0.00154923	up
Mmp8	chr9:7558428–7568486	19.994	1.48647	13.45070606	0.00005	0.00154923	up
Hspa1b	chr17:34956435–34959819	19.5097	1.61536	12.07766787	0.00005	0.00154923	up
Gdf15	chr8:70629392–70632456	4.13844	0.369078	11.21291907	0.00005	0.00154923	up
Ereg	chr5:91051870–91093649	0.895565	0.0798815	11.2111316	0.00005	0.00154923	up
Hmox1	chr8:75093590–75100596	116.38	10.8181	10.75794043	0.00005	0.00154923	up
Pram1	chr17:33629089–33645706	4.03205	0.466964	8.634603914	0.00225	0.0357798	up
Hspa1a	chr17:34969198–34972154	8.92662	1.05997	8.421574586	0.00005	0.00154923	up
Cyp26b1	chr6:84571413–84593908	87.0178	10.6216	8.19253539	0.00005	0.00154923	up
Tmc3	chr7:83584926–83631842	1.44366	0.182715	7.901135484	0.00005	0.00154923	up
Gsg1l	chr7:125878418–126082411	0.0516177	0.838534	-16.245069	0.00115	0.0211396	down
Ankle1	chr8:71406009–71409904	0.141143	1.42103	-10.068033	0.00005	0.0015492	down
Shisa2	chr14:59625280–59631658	0.333449	2.54505	-7.6324699	0.00005	0.0015492	down
Pbk	chr14:65805836–65817822	0.549558	3.86568	-7.034169	0.00005	0.0015492	down
Tex14	chr11:87405064–87555823	0.266288	1.81074	-6.7999305	0.01255	0.128151	down
Gmnc	chr16:26957235–26989974	0.294189	1.97052	-6.6981375	0.00005	0.0015492	down
Fam83a	chr15:57,985418–58011009	0.137421	0.906131	-6.5938451	0.0038	0.0527118	down
Kntc1	chr5:123749725–123821593	0.179585	1.1453	-6.3774956	0.00005	0.0015492	down
Ckap2	chr8:22168151–22185819	0.73018	4.64288	-6.3585596	0.00005	0.0015492	down
4933403O08Rik	chrX:112239048–112243852	0.15683	0.996082	-6.3513356	0.0223	0.186433	down
Lrrc17	chr5:21483846–21645605	0.483547	3.02097	-6.2475043	0.0001	0.0028309	down
Slc10a5	chr3:10331733–10335656	0.455844	2.59509	-5.6929246	0.00005	0.0015492	down
4930578C19Rik	chrX:18414880–18461397	0.926166	5.0221	-5.4224694	0.00015	0.0040133	down
Ces2e	chr8:104926259–104934672	0.415766	2.23277	-5.3702528	0.00005	0.0015492	down
Prss35	chr9:86743632–86758272	0.174285	0.900453	-5.1665456	0.0003	0.007291	down
Fam64a	chr11:72042031–72047370	0.458055	2.32908	-5.0846906	0.00045	0.0101309	down
5830454E08Rik	chr9:120577330–120578073	0.202576	1.01435	-5.0072552	0.02245	0.187151	down
Hist1h2bp	chr13:21787460–21789213	0.420843	2.10283	-4.9966806	0.01565	0.148535	down
Cyp2ab1	chr16:20308386–20323065	0.551711	2.70829	-4.9089008	0.00005	0.0015492	down
Ccdc129	chr6:55836894–55978735	1.18027	5.68789	-4.8191209	0.00005	0.0015492	down

### GO and KEGG analyses of DE mRNAs

**Fig. 5:**
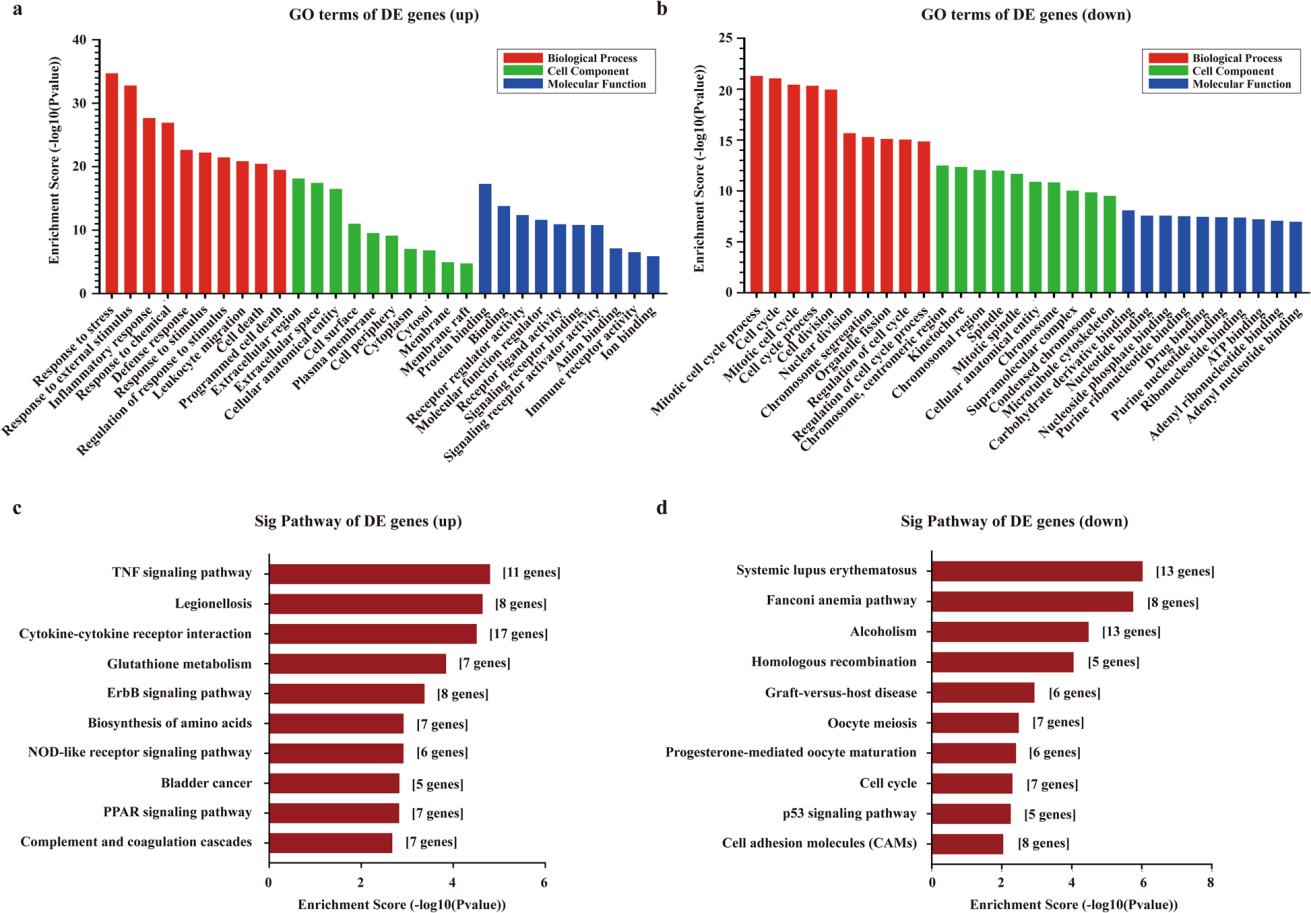
GO and KEGG analyses of DE mRNAs. **a-b** GO analysis. **a** up-regulated, **b** down-regulated. **c-d** KEGG signaling pathway analysis of DE mRNAs. **c** up-regulated, **d** down-regulated

To investigate the potential function of the mRNAs, GO and KEGG pathway enrichment analyses of the DE mRNAs were performed. The GO analysis results, including BP, CC and MF categories are shown. For the up-regulated mRNAs, response to stress, extracellular region and protein binding are the most enriched GO terms in BP, CC and MF categories, respectively (Fig. 5a). The most enriched GO terms among the down-regulated mRNAs are mitotic cell cycle process in the BP category, chromosome, centromeric region in the CC category and carbohydrate derivative binding in the MFs category (Fig. 5b). KEGG pathway analysis indicated that the up-regulated mRNAs might be involved in the TNF signaling pathway and cytokine-cytokine receptor interaction (Fig. 5c). For the down-regulated mRNAs, the Fanconi anemia pathway and homologous recombination might play an essential role in PBLI (Fig. 5d).

### PPI network, molecular complex detection analysis and hub gene identification

**Fig. 6:**
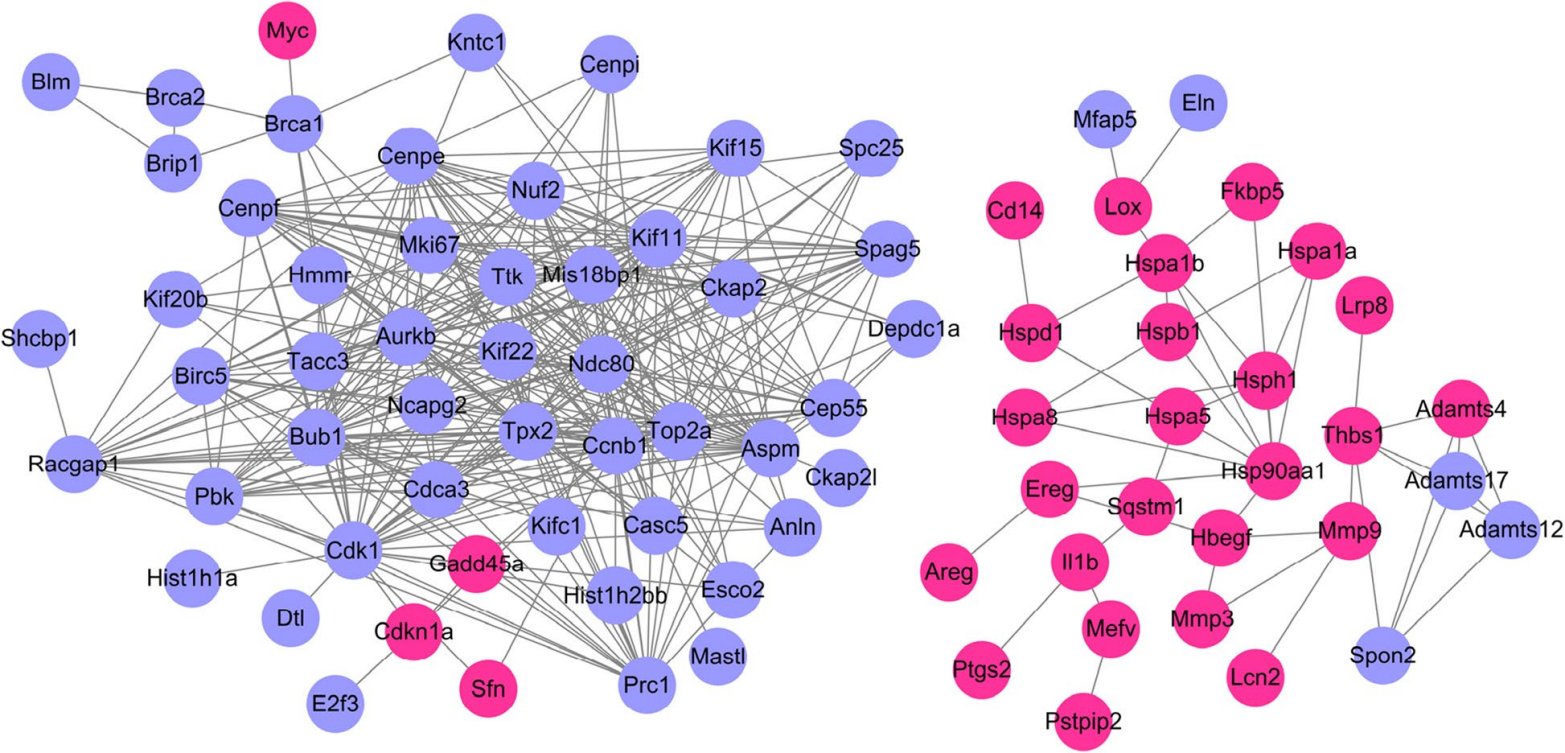
PPI network of the differentially expressed mRNAs. Purple represents downregulated mRNAs and pink represents up-regulated mRNAs

**Fig. 7:**
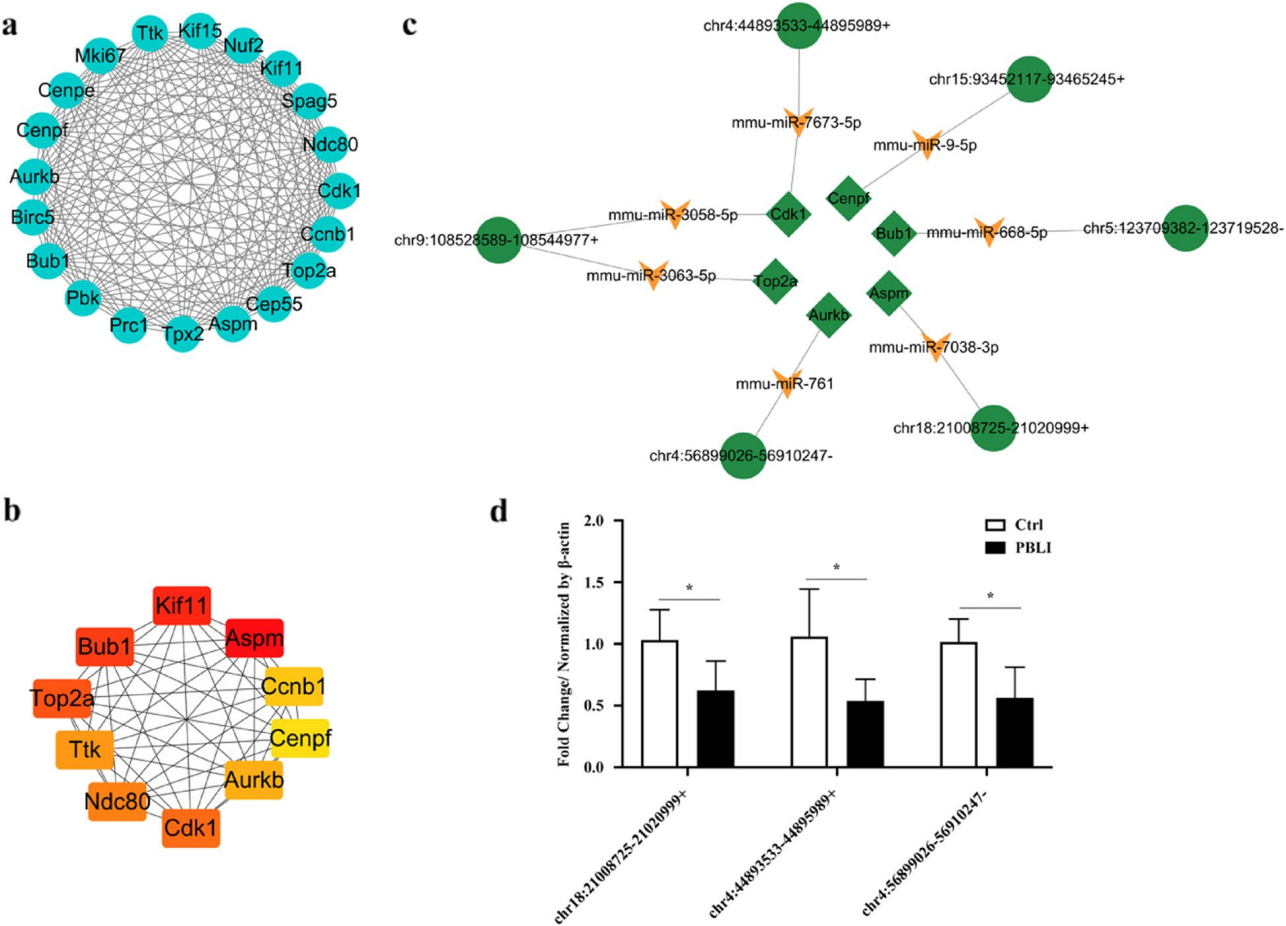
Hub gene identification and construction of a circRNA-miRNA-hub gene network. **a** Selected module of the PPI network. **b** Hub gene network. **c** circRNA-miRNA-hub gene network. The triangle, quadrilateral and circle represent miRNA, mRNA and circRNA, respectively. **d** Validation of circRNAs in circRNA-miRNA-hub gene network by qRT-PCR. The results are presented as the mean ± SD. *P < 0.05

A PPI network including 234 nodes and 717 edges was built for the 681 DE mRNAs using the STRING database (Fig. 6). Subsequently, we used the MCODE method to screen hub genes from the PPI network, and the first module was selected after MCODE analysis (Fig. 7a). According to the MCC algorithm, the top 10 genes in the PPI network were obtained as hub genes, including Cenpf, Ndc80, Cdk1, Aurkb, Ttk, Aspm, Ccnb1, Kif11, Bub1 and Top2a (Fig. 7b), which are all down-regulated.

### Construction of a circRNA-miRNA-hub gene network

To construct the circRNA-miRNA-hub gene network, hub gene-related miRNAs, and top 20 DE circRNAs targeted miRNAs were predicted. As shown in Fig. 7c, the network consist of 6 circRNAs (chr18:21008725–21020999 + , chr4:44893533–44895989 + , chr4:56899026–56910247-, chr5:123709382–123719528-, chr9:108528589–108544977 + and chr15:93452117–93465245 +), 7 miRNAs (mmu-miR-3058-5p, mmu-miR-3063-5p, mmu-miR-668-5p, mmu-miR-7038-3p, mmu-miR-761, mmu-miR-7673-5p and mmu-miR-9-5p) and 6 mRNAs (Aspm, Aurkb, Bub1, Cdk1, Cenpf and Top2a) with 18 nodes and 14 edges was constructed. And chr18:21008725–21020999 + , chr4:44893533–44895989 + and chr4:56899026–56910247- were randomly selected for validation of circRNAs in the network. The results showed significant changes in the expression of these circRNAs in the lung tissue of mice exposed to the blast wave (Fig. 7d).

## Discussion

Explosions can cause severe internal pulmonary injury despite tiny injuries observed on the external chest. This makes it difficult for PBLI to be diagnosed early. Therefore, early diagnosis and treatment are particularly important for victims suffering from PBLI [27]. Currently, PBLI is diagnosed with clinical manifestations such as hypoxemia and respiratory dysfunction, combined with chest X-ray and chest CT examination [28, 29], which are commonly performed in hospitals instead of rescue sites. In addition, although many studies and therapeutic trials have been conducted, at the present stage, the treatment of PBLI is predominantly supportive and symptomatic [6]. Due to the complexity and difficulty in diagnosis and treatment, the mortality rate of PBLI is extremely high. Therefore, the development of rapid diagnosis and specific treatment strategies is particularly important for reducing mortality. In this study, we constructed a circRNA-miRNA-hub gene network to explore the role of circRNAs in PBLI, aiming to provide new ideas for the PBLI diagnosis and treatment.

CircRNAs are a unique class of non-coding RNAs, as they are more abundant, more stable, and have certain tissue specificity than other types of RNA. CircRNAs are considered to be promising biomarkers and potential therapeutic targets of many diseases. CircRNA has been reported to be associated with a variety of diseases. For example, Wang et al., reviewed circRNAs that associated with cancers, including gastric cancer, breast cancer, lung cancer and pancreatic cancers [12]. Yang et al., demonstrated that circ_0054633 is highly expressed in lipopolysaccharides (LPS)-induced acute lung injury model both in vivo and in vitro. Silencing of circ_0054633 alleviates LPS-induced ALI via the NF-κB signaling pathway, suggesting that circ_0054633 may be a potential biomarker for the diagnosis and treatment of ALI [30]. In this study, the DE circRNAs in PBLI were selected. The changes in the expression of these circRNAs induced by PBLI indicate that these circRNAs may be involved in PBLI. Which provides a basis for the screening of PBLI diagnostic and treatment markers.

There is growing evidence that circRNAs can act as ceRNA by sponging miRNA by mitigating the inhibition of miRNAs on their target. As the mechanism of PBLI is still unclear, the circRNA related to PBLI is basically not studied, the correlation between circRNA and PBLI is unknown, and the corresponding PBLI database and circRNA-PBLI association prediction methods are lacking. The application of bioinformatics methods to predict DE circRNA-targeted miRNAs and construct ceRNA networks is crucial for screening possible circRNAs and inferring the possible mechanism of action of circRNAs in PBLI. In this study, the circRNA-miRNA-hub gene network consisting of 6 circRNAs, 7 miRNAs and 6 mRNAs was constructed. These circRNA-targeted mRNAs play an important role in inflammation and oxidative stress, which is responsible for the high mortality rate of PBLI [31, 32]. For example, it has been reported that in hepatocellular carcinoma, the expression levels of CDK1 was positively correlated with the infiltration levels of CD4^+^ T cells, CD8^+^ T cells, neutrophils, macrophages and dendritic cells, suggesting that this gene may be involved in the recruitment and regulation of infiltrating cells in the immune microenvironment [33]. And in Bleomycin-induced ALI, the protein expression of Top2a was up-regulated, which leads to persistent inflammation and aggravation of alveolar epithelium [34]. Thus, these genes may be involved in immune regulation and inflammation balance in PBLI. And the 6 circRNAs (chr18:21008725–21020999 + , chr4:44893533–44895989 + , chr4:56899026–56910247-, chr5:123709382–123719528-, chr9:108528589–108544977 + and chr15:93452117–93465245 +) that in the circRNA-miRNA-hub gene network may also play important regulatory roles in the progression of PBLI, and may be used as targets for the diagnosis and treatment of PBLI.

As mentioned above, we used bioinformatics methods to explore PBLI-associated circRNAs. At present, more and more computational methods have been widely used in the field of bioinformatics, and computational models can effectively predict potential circRNA-disease associations, which is time-saving and inexpensive. These computational models can be broadly divided into two categories, those based on network algorithms and those based on machine learning [12]. For example, Zhao et al. developed the computational method of IBNPKAT and Ge et al., developed LLCDC [35, 36]. These methods obtain circRNA information, disease information and circRNA-disease associations from the database, and use circRNA similarity and disease similarity to infer potential circRNA-disease relationship. Case studies show that these methods are effective in predicting circRNAs associated with human disease and do not require negative samples. With the help of these computational models, the correlation between circRNAs and disease can be quickly deduced and potential circRNAs can be screened. In addition to circRNAs, computational models successfully developed for other research areas will significantly accelerate the prediction of PBLI related ncRNAs. For instance, based on network distance analysis model (NDALMA), and graph convolutional neural network and conditional random field model (GCNCRF) for lncRNA-miRNA association prediction, as well as based on deep learning predictive model (DMFGAM) for predicting drug-target interactions [37–39]. It is believed that these computational biology methods will provide new insights into the molecular mechanism and treatment strategies of PBLI in the future.

## Conclusions

In conclusion, this study delineated the expression profiles of circRNAs and constructed circRNA-miRNA-hub gene network in PBLI for the first time, which may provide new ideas for the diagnosis and treatment of PBLI.

## Data Availability

The transcriptome high throughput sequencing data were deposited in GEO with accession number of GSE195726 (https://www.ncbi.nlm.nih.gov/geo/query/acc.cgi?acc=GSE195726) Enter token sxkpeqiujlybdcj into the box.

## References

[1] Mathews ZR, Koyfman A (2015). Blast Injuries. J Emerg Med.

[2] Junuzovic M (2022). Explosion fatalities in Sweden, 2000–2018. Med Sci Law.

[3] Hua W, Chen J, Qin Q, Wan Z, Song L (2021). Causation analysis and governance strategy for hazardous cargo accidents at ports: case study of Tianjin Port's hazardous cargo explosion accident. Mar Pollut Bull.

[4] Cheng TM, Lin Y, Gu DQ, Luo CK, Zheng HE (1984). Ultrastructural changes of bone marrow megakaryocytes in several types of injury. Burns Incl Therm Inj.

[5] Qi XL, Hao J, Huang LJ, Wu S, Ma HH, Ye ZQ, He HB, Li SW, Li CE, Huang X (2018). Apoptotic mechanisms in rabbits with blast-induced acute lung injury 1. Acta Cir Bras.

[6] Chen K, Yang J, Xiao F, Chen J, Hu W, Wang X, Wang L, Du J, Jiang J, He Y (2020). Early peritoneal dialysis ameliorates blast lung injury by alleviating pulmonary edema and inflammation. Shock.

[7] Mishra SK, Kumar BS, Khushu S, Singh AK, Gangenahalli G (2017). Early monitoring and quantitative evaluation of macrophage infiltration after experimental traumatic brain injury: a magnetic resonance imaging and flow cytometric analysis. Mol Cell Neurosci.

[8] Wang H, Zhang WJ, Gao JH, Liu JR, Liu ZY, Xia BQ, Fan XL, Li CZ, Qian AR (2020). Global gene expression profiling of blast lung injury of goats exposed to shock wave. Chin J Traumatol.

[9] Yang C, Dong-Hai Z, Ling-Ying L, Yong-Hui Y, Yang W, Li-Wei Z, Rui-Guo H, Jia-Ke C (2019). Simulation of blast lung injury induced by shock waves of five distances based on finite element modeling of a three-dimensional rat. Sci Rep.

[10] Altesha MA, Ni T, Khan A, Liu K, Zheng X (2019). Circular RNA in cardiovascular disease. J Cell Physiol.

[11] Ye Y, Zhang L, Hu T, Yin J, Xu L, Pang Z, Chen W (2021). CircRNA_103765 acts as a proinflammatory factor via sponging miR-30 family in Crohn's disease. Sci Rep.

[12] Wang CC, Han CD, Zhao Q, Chen X (2021). Circular RNAs and complex diseases: from experimental results to computational models. Brief Bioinform.

[13] Kristensen LS, Andersen MS, Stagsted LVW, Ebbesen KK, Hansen TB, Kjems J (2019). The biogenesis, biology and characterization of circular RNAs. Nat Rev Genet.

[14] Tay Y, Rinn J, Pandolfi PP (2014). The multilayered complexity of ceRNA crosstalk and competition. Nature.

[15] Gene Ontology Consortium. Gene Ontology Consortium: going forward. Nucleic Acids Res. 2015;43(Database issue):D1049–1056. 10.1093/nar/gku1179 PMC4383973 25428369

[16] Kanehisa M, Sato Y, Kawashima M, Furumichi M, Tanabe M (2016). KEGG as a reference resource for gene and protein annotation. Nucleic Acids Res.

[17] Jin X, Feng CY, Xiang Z, Chen YP, Li YM (2016). CircRNA expression pattern and circRNA-miRNA-mRNA network in the pathogenesis of nonalcoholic steatohepatitis. Oncotarget.

[18] Deng Y, Song H, Xiao Y, Zhao Y, Chu L, Ding J, Shen X, Qi X (2022). High-Throughput Sequencing to Investigate lncRNA-circRNA-miRNA-mRNA Networks Underlying the Effects of Beta-Amyloid Peptide and Senescence on Astrocytes. Front Genet.

[19] Su D, Huang Y, Liu D, Huang Y, Ye B, Qin S, Chen C, Pang Y (2022). Bioinformatic analysis of dysregulated circular RNAs in pediatric pulmonary hypertension linked congenital heart disease. Transl Pediatr.

[20] Meng XY, Lu QY, Zhang JF, Li JF, Shi MY, Huang SY, Yu SF, Zhao YM, Fan HJ (2022). A novel animal model of primary blast lung injury and its pathological changes in mice. J Trauma Acute Care Surg.

[21] Kanehisa M, Goto S (2000). KEGG: kyoto encyclopedia of genes and genomes. Nucleic Acids Res.

[22] Kanehisa M (2019). Toward understanding the origin and evolution of cellular organisms. Protein Sci.

[23] Kanehisa M, Furumichi M, Sato Y, Kawashima M, Ishiguro-Watanabe M (2023). KEGG for taxonomy-based analysis of pathways and genomes. Nucleic Acids Res.

[24] Chen S, Zhang Y, Ding X, Li W (2022). Identification of lncRNA/circRNA-miRNA-mRNA ceRNA Network as Biomarkers for Hepatocellular Carcinoma. Front Genet.

[25] Xiong DD, Dang YW, Lin P, Wen DY, He RQ, Luo DZ, Feng ZB, Chen G (2018). A circRNA-miRNA-mRNA network identification for exploring underlying pathogenesis and therapy strategy of hepatocellular carcinoma. J Transl Med.

[26] Liu X, Zeng Y, Liu Z, Li W, Wang L, Wu M (2022). Bioinformatics analysis of the circRNA-miRNA-mRNA network for atrial fibrillation. Medicine (Baltimore).

[27] Xue YQ, Wu CS, Zhang HC, Du J, Sun JH, Zhang AQ, Zeng L, Zhang M, Jiang JX (2020). Value of lung ultrasound score for evaluation of blast lung injury in goats. Chin J Traumatol.

[28] Scott TE, Kirkman E, Haque M, Gibb IE, Mahoney P, Hardman JG (2017). Primary blast lung injury - a review. Br J Anaesth.

[29] Peng LH, Guo GH (2016). Advances in the research of blast lung injury. Zhonghua Shao Shang Za Zhi.

[30] Yang CL, Yang WK, He ZH, Guo JH, Yang XG, Li HB (2021). Quietness of circular RNA circ_0054633 alleviates the inflammation and proliferation in lipopolysaccharides-induced acute lung injury model through NF-κB signaling pathway. Gene.

[31] Smith JE (2011). The epidemiology of blast lung injury during recent military conflicts: a retrospective database review of cases presenting to deployed military hospitals, 2003–2009. Philos Trans R Soc Lond B Biol Sci.

[32] Smith JE (2011). Blast lung injury. J R Nav Med Serv.

[33] Zou Y, Ruan S, Jin L, Chen Z, Han H, Zhang Y, Jian Z, Lin Y, Shi N, Jin H (2020). CDK1, CCNB1, and CCNB2 are Prognostic Biomarkers and Correlated with Immune Infiltration in Hepatocellular Carcinoma. Med Sci Monit.

[34] Shaikh SB, Najar MA, Prabhu A, Rex DAB, Chanderasekaran J, Behera SK, Modi PK, Prasad TSK, Bhandary YP (2021). The unique molecular targets associated antioxidant and antifibrotic activity of curcumin in in vitro model of acute lung injury: A proteomic approach. BioFactors.

[35] Zhao Q, Yang Y, Ren G, Ge E, Fan C (2019). Integrating Bipartite Network Projection and KATZ Measure to Identify Novel CircRNA-Disease Associations. IEEE Trans Nanobioscience.

[36] Ge E, Yang Y, Gang M, Fan C, Zhao Q (2020). Predicting human disease-associated circRNAs based on locality-constrained linear coding. Genomics.

[37] Zhang L, Yang P, Feng H, Zhao Q, Liu H (2021). Using Network Distance Analysis to Predict lncRNA-miRNA Interactions. Interdiscip Sci.

[38] Wang W, Zhang L, Sun J, Zhao Q, Shuai J (2022). Predicting the potential human lncRNA-miRNA interactions based on graph convolution network with conditional random field. Brief Bioinform.

[39] Wang T, Sun J, Zhao Q (2023). Investigating cardiotoxicity related with hERG channel blockers using molecular fingerprints and graph attention mechanism. Comput Biol Med.

